# A quantitative theory of the functions of the hippocampal CA3 network in memory

**DOI:** 10.3389/fncel.2013.00098

**Published:** 2013-06-25

**Authors:** Edmund T. Rolls

**Affiliations:** ^1^Oxford Centre for Computational NeuroscienceOxford, UK; ^2^Department of Computer Science, University of WarwickCoventry, UK

**Keywords:** hippocampus, attractor network, competitive network, episodic memory, spatial view neurons, object-place memory, recall, pattern separation

## Abstract

A quantitative computational theory of the operation of the hippocampal CA3 system as an autoassociation or attractor network used in episodic memory system is described. In this theory, the CA3 system operates as a single attractor or autoassociation network to enable rapid, one-trial, associations between any spatial location (place in rodents, or spatial view in primates) and an object or reward, and to provide for completion of the whole memory during recall from any part. The theory is extended to associations between time and object or reward to implement temporal order memory, also important in episodic memory. The dentate gyrus (DG) performs pattern separation by competitive learning to produce sparse representations suitable for setting up new representations in CA3 during learning, producing for example neurons with place-like fields from entorhinal cortex grid cells. The dentate granule cells produce by the very small number of mossy fiber (MF) connections to CA3 a randomizing pattern separation effect important during learning but not recall that separates out the patterns represented by CA3 firing to be very different from each other, which is optimal for an unstructured episodic memory system in which each memory must be kept distinct from other memories. The direct perforant path (pp) input to CA3 is quantitatively appropriate to provide the cue for recall in CA3, but not for learning. Tests of the theory including hippocampal subregion analyses and hippocampal NMDA receptor knockouts are described, and support the theory.

## Introduction

In this paper a computational theory of how the hippocampal CA3 region operates and is involved in episodic memory is described. I consider how the network architecture of the hippocampal CA3 system may enable it to contribute to episodic memory, and to recall the whole of a memory when a partial retrieval cue is present using the recurrent collateral connections which I argue implement an autoassociation or attractor memory. I focus on a fundamental property of episodic memory, the ability to store and retrieve the memory of a particular single event involving an association between items such as the place and the object or reward seen at that place. Episodic memory in the sense of a series of linked events requires this type of event memory, and could be implemented by linking together a series of events. After the theory is presented, I then describe neurophysiological and related evidence that tests the theory. New hypotheses are described about the advantages of the diluted connectivity present in the CA3 network. The effects of the diluted connectivity of the CA3 recurrent collateral network, and the graded firing rates of hippocampal neurons, on the noise due to the randomness of the spiking times of CA3 neurons, and thus on the operation of an attractor system such as the CA3 network, are described. A hypothesis about why the connectivity in cortical networks is proposed. Factors that influence the stability of the CA3-CA3 recurrent collateral network are also considered, together with their possible implications for neuropsychiatric conditions such as schizophrenia.

Episodic memory, the memory of a particular episode, requires the ability to remember particular events, and to distinguish them from other events. An event consists of a set of items that occur together, such as seeing a particular object or person's face in a particular place. An everyday example might be remembering where one was for dinner, who was present, what was eaten, what was discussed, and the time at which it occurred. The spatial context is almost always an important part of an episodic memory (Dere et al., 2008), and it may be partly for this reason that episodic memory is linked to the functions of the hippocampal system, which is involved in spatial processing and memory.

In this paper I also show that the primate hippocampus including CA3 has a special representation of space that makes it particularly appropriate for episodic memory in primates including humans, for it is a representation of space “out there.” This enables memories to be formed of what one has seen at a particular place, even if one has not been to the place. This is not possible with rodent place cells, which respond to the place where the rodent is located. I also show that the primate hippocampus has more than only a spatial representation, for it also represents objects that are seen at particular places, and rewards that are found at particular places in spatial scenes. I also show that primate hippocampal neurons are activated when a memory must be recalled from a part of a memory, for example when the place at which an object was shown must be recalled when the object is seen alone. The ability to recall a whole memory from a partial cue is an important property of episodic memory.

## Systems-level functions and connections of the primate hippocampus

Any theory of the hippocampus must state at the systems level what is computed by the hippocampus. Some of the relevant evidence about the functions of the hippocampus in memory comes from the effects of damage to the hippocampus, the responses of neurons in the hippocampus during behavior, and the systems-level connections of the hippocampus, described in more detail elsewhere (Rolls and Kesner, 2006; Rolls and Xiang, 2006; Rolls, 2008, 2010b). Many of the memory functions are important in event or episodic memory, in which the ability to remember what happened where on typically a single occasion (or trial in a learning experiment) is important. It will be suggested below that an autoassociation memory implemented by the CA3 neurons enables event or episodic memories to be formed by enabling associations to be formed between spatial and other including object or reward representations.

**Figure 1:**
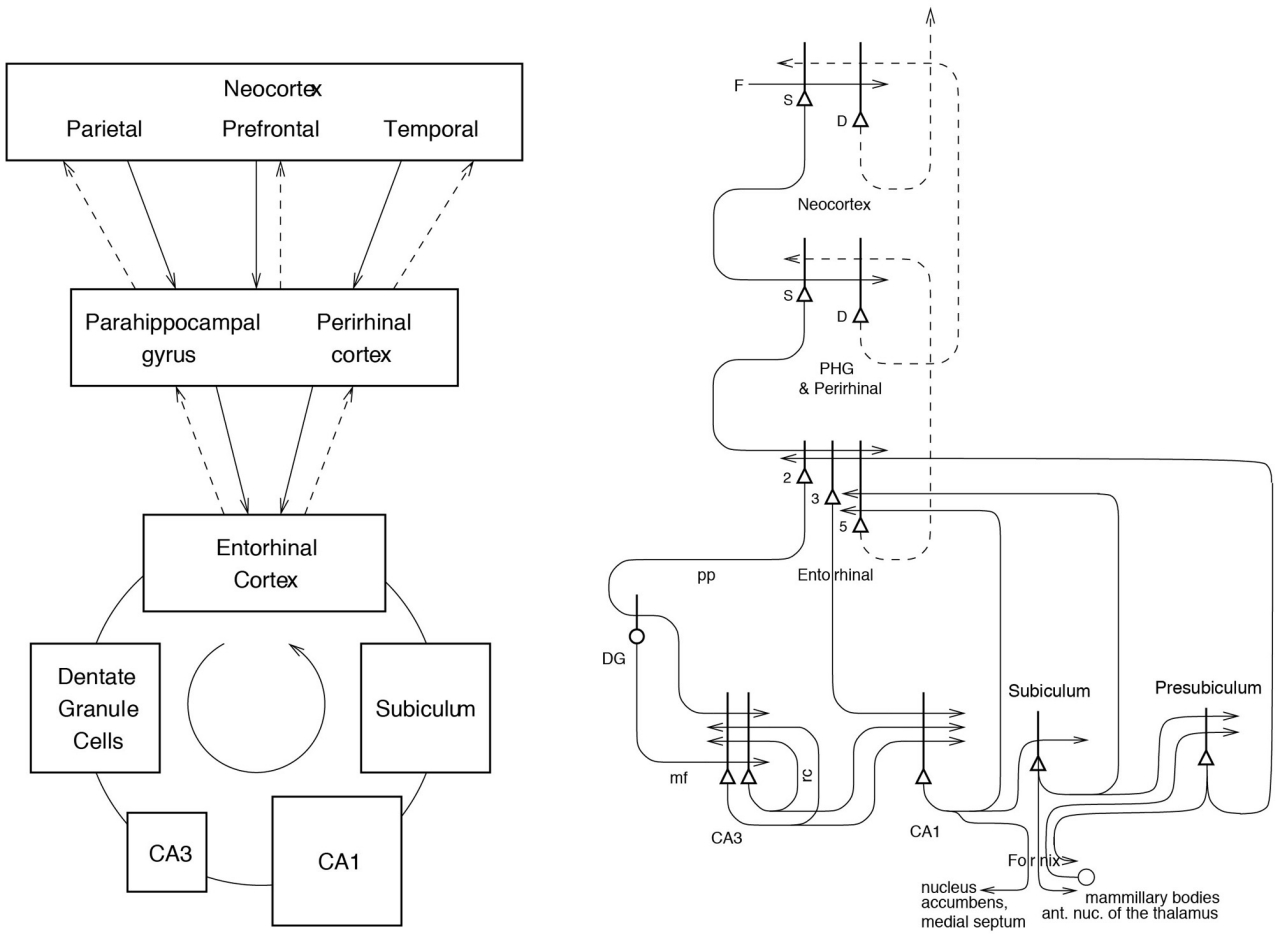
**Forward connections (solid lines) from areas of cerebral association neocortex via the parahippocampal gyrus and perirhinal cortex, and entorhinal cortex, to the hippocampus; and backprojections (dashed lines) via the hippocampal CA1 pyramidal cells, subiculum, and parahippocampal gyrus to the neocortex**. There is great convergence in the forward connections down to the single network implemented in the CA3 pyramidal cells; and great divergence again in the backprojections. **Left:** block diagram. **Right:** more detailed representation of some of the principal excitatory neurons in the pathways. Abbreviations: D, Deep pyramidal cells; DG, Dentate Granule cells; F, Forward inputs to areas of the association cortex from preceding cortical areas in the hierarchy; mf, mossy fibers; PHG, parahippocampal gyrus and perirhinal cortex; pp, perforant path; rc, recurrent collateral of the CA3 hippocampal pyramidal cells; S, Superficial pyramidal cells; 2, pyramidal cells in layer 2 of the entorhinal cortex; 3, pyramidal cells in layer 3 of the entorhinal cortex; 5, pyramidal cells in layer 5 of the entorhinal cortex. The thick lines above the cell bodies represent the dendrites.

Information stored in the hippocampus will need to be retrieved and affect other parts of the brain in order to be used. The information about episodic events recalled from the hippocampus could be used to help form semantic memories (Rolls, 1989b,c, 1990a; Treves and Rolls, 1994). For example, remembering many particular journeys could help to build a geographic cognitive map in the neocortex. The hippocampus and neocortex would thus be complementary memory systems, with the hippocampus being used for rapid, “on the fly,” unstructured storage of information involving activity potentially arriving from many areas of the neocortex; while the neocortex would gradually build and adjust on the basis of much accumulating information, often recalled from the hippocampal unstructured store, the semantic representation (Rolls, 1989b; Treves and Rolls, 1994; McClelland et al., 1995; Moscovitch et al., 2005). The theory described below shows how information could be retrieved within the hippocampus, and how this retrieved information could enable the activity in neocortical areas that was present during the original storage of the episodic event to be reinstated, thus implementing recall, by using hippocampo-neocortical backprojections as described elsewhere (Treves and Rolls, 1994; Rolls, 1995, 1996b, 2008, 2010b) (see Figure 1).

To understand the functions of the primate hippocampus in event or episodic memory, it is necessary to understand which other parts of the brain it receives information from. Does it for example receive object as well as spatial information in terms of its connectivity? The primate hippocampus receives inputs via the entorhinal cortex (area 28) and the highly developed parahippocampal gyrus (areas TF and TH) as well as the perirhinal cortex from the ends of many processing streams of the cerebral association cortex, including the visual and auditory temporal lobe association cortical areas, the prefrontal cortex, and the parietal cortex (Van Hoesen, 1982; Amaral, 1987; Amaral et al., 1992; Suzuki and Amaral, 1994b; Witter et al., 2000; Lavenex et al., 2004; Rolls and Kesner, 2006; Rolls, 2008) (see Figure 1). The hippocampus is thus by its connections potentially able to associate together object and spatial representations. In addition, the entorhinal cortex receives inputs from the amygdala, and the orbitofrontal cortex, which could provide reward-related information to the hippocampus (Suzuki and Amaral, 1994a; Carmichael and Price, 1995; Stefanacci et al., 1996; Pitkanen et al., 2002).

The primary output from the hippocampus to neocortex originates in CA1 and projects to subiculum, entorhinal cortex, and parahippocampal structures (areas TF-TH) as well as prefrontal cortex (Van Hoesen, 1982; Witter, 1993; Delatour and Witter, 2002; Van Haeften et al., 2003) (see Figure 1), though there are other outputs (Rolls and Kesner, 2006). These are the pathways that are likely to be involved in the recall of information from the hippocampus.

## A theory of the operation of the hippocampal CA3 circuitry as part of a memory system

In this section, I consider how event or episodic memories might be learned and retrieved by hippocampal circuitry, and in addition retrieved back into the neocortex. The theory has been developed through many stages (Rolls, 1987, 1989a,b,c, 1990a,b, 1991, 1995, 1996b, 2008, 2010b; Treves and Rolls, 1991, 1992, 1994; Rolls and Treves, 1998; Rolls and Kesner, 2006; Rolls and Deco, 2010), has as a predecessor developments made by Marr (1971; though he never identified the CA3 system as an autoassociation network), and has benefitted greatly from collaborations with many whose names appear below in the citations, including Alessandro Treves and Simon Stringer.

### Hippocampal circuitry

**Figure 2:**
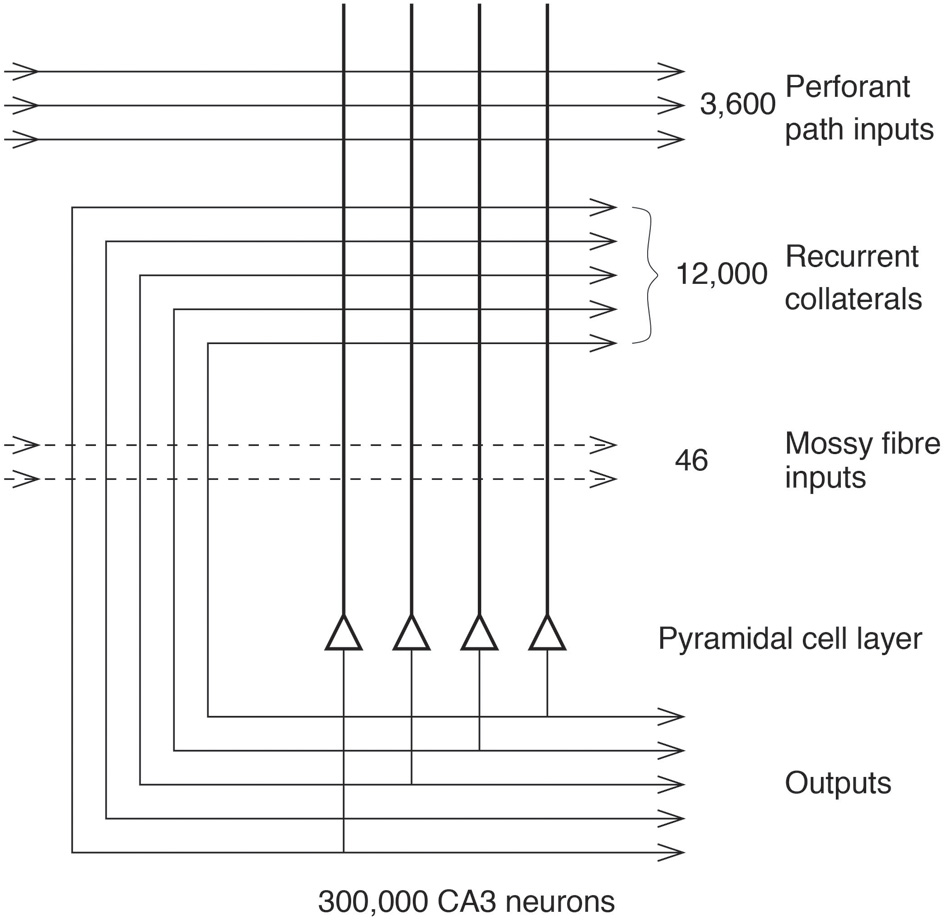
**The numbers of connections from three different sources onto each CA3 cell from three different sources in the rat (After Treves and Rolls, 1992; Rolls and Treves, 1998)**.

Projections from the entorhinal cortex layer 2 reach the granule cells (of which there are 10^6^ in the rat) in the dentate gyrus (DG), via the perforant path (pp; Witter, 1993). The granule cells project to CA3 cells via the mossy fibers (MFs), which provide a *sparse* but possibly powerful connection to the 3·10^5^ CA3 pyramidal cells in the rat. Each CA3 cell receives ~46 MF inputs, so that the sparseness of this connectivity is thus 0.005%. By contrast, there are many more—possibly weaker—direct pp inputs also from layer 2 of the entorhinal cortex onto each CA3 cell, in the rat of the order of 4·10^3^. The largest number of synapses (about 1.2·10^4^ in the rat) on the dendrites of CA3 pyramidal cells is, however, provided by the (recurrent) axon collaterals of CA3 cells themselves (rc; see Figure 2). It is remarkable that the recurrent collaterals are distributed to other CA3 cells largely throughout the hippocampus (Amaral and Witter, 1989, 1995; Amaral et al., 1990; Ishizuka et al., 1990; Witter, 2007), so that effectively the CA3 system provides a single network, with a connectivity of ~2% between the different CA3 neurons given that the connections are bilateral. The CA3-CA3 recurrent collateral system is even more extensive in macaques than in rats (Kondo et al., 2009). The neurons that comprise CA3, in turn, project to CA1 neurons via the Schaffer collaterals. In addition, projections that terminate in the CA1 region originate in layer 3 of the entorhinal cortex (see Figure 1 and Amaral and Witter, 1989; Storm-Mathiesen et al., 1990; Amaral, 1993; Witter et al., 2000; Naber et al., 2001; Lavenex et al., 2004; Andersen et al., 2007; Witter, 2007; Kondo et al., 2009).

### CA3 as an autoassociation or attractor memory

#### Arbitrary associations, and pattern completion in recall

Many of the synapses in the hippocampus show associative modification as shown by long-term potentiation, and this synaptic modification appears to be involved in learning (see Morris, 1989, 2003; Morris et al., 2003; Nakazawa et al., 2003, 2004; Lynch, 2004; Andersen et al., 2007; Wang and Morris, 2010). On the basis of the evidence summarized above, Rolls (1987, 1989a,b,c, 1990a,b,^1991^) and others (McNaughton and Morris, 1987; Levy, 1989; McNaughton, 1991) have suggested that the CA3 stage acts as an autoassociation memory which enables episodic memories to be formed and stored in the CA3 network, and that subsequently the extensive recurrent collateral connectivity allows for the retrieval of a whole representation to be initiated by the activation of some small part of the same representation (the cue). The crucial synaptic modification for this is in the recurrent collateral synapses. [A description of the operation of autoassociative networks is provided in detail elsewhere (Amit, 1989; Hertz et al., 1991; Rolls and Treves, 1998; Rolls and Deco, 2002, 2010; Rolls, 2010a) including *Memory, Attention, and Decision-Making* (Rolls, 2008)].

**Figure 3:**
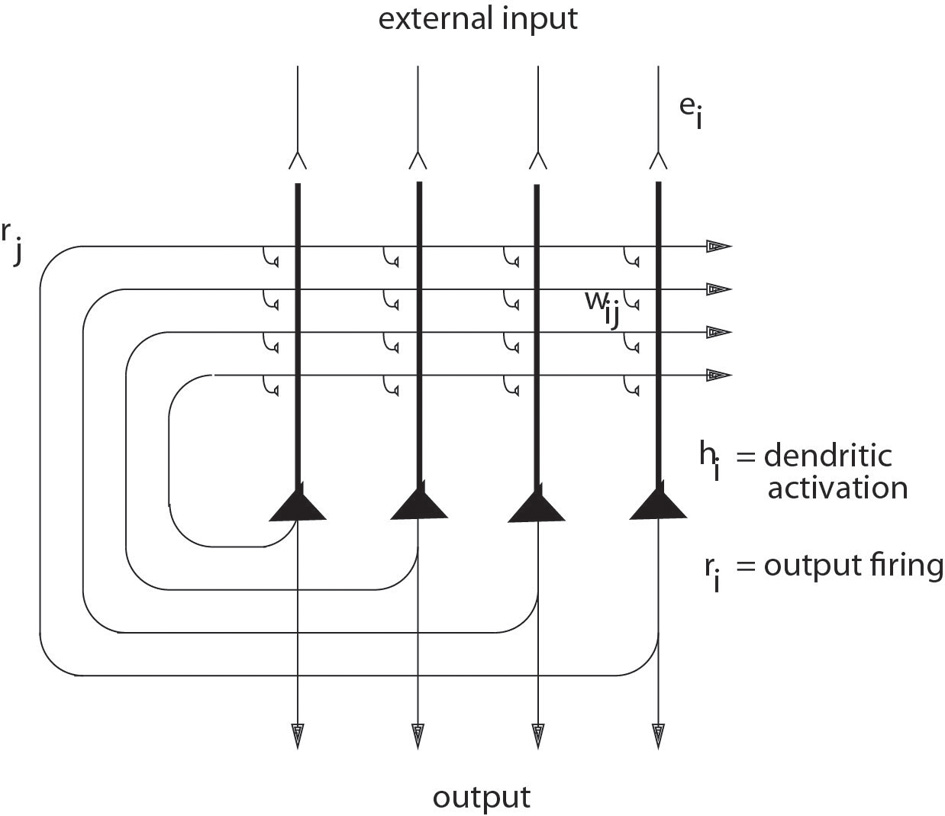
**The architecture of an autoassociation or attractor neural network (CANN) (see text)**.

The architecture of an autoassociation network is shown in Figure 3, and the learning rule for the change in the synaptic weights is as shown in Equation 1 (Rolls and Treves, 1998; Rolls and Deco, 2002; Rolls, 2008).

(1) $$\delta w_{ij}=k\cdot r_{i}\cdot {r^{\prime }}_{j}$$

where *k* is a constant, *r*_*i*_ is the activation of the dendrite (the postsynaptic term), *r*′_*j*_ is the presynaptic firing rate, and δ *w*_*ij*_ is the change in the synaptic weight *w*_*ij*_. [*w*_*ij*_ refers to the *j*'th synapse onto the *i*'th neuron. An introduction to autoassociation, competitive, and pattern association networks is provided in the Appendices of *Memory*, *Attention*, *and Decision-Making: A Unifying Computational Neuroscience Approach* (Rolls, 2008)].

The hypothesis is that because the CA3 operates effectively as a single network, it can allow arbitrary associations between inputs originating from very different parts of the cerebral cortex to be formed. These might involve associations between information originating in the temporal visual cortex about the presence of an object, and information originating in the parietal cortex about where it is. I note that although there is some spatial gradient in the CA3 recurrent connections, so that the connectivity is not fully uniform (Ishizuka et al., 1990; Witter, 2007), nevertheless the network will still have the properties of a single interconnected autoassociation network allowing associations between arbitrary neurons to be formed, given the presence of many long-range connections which overlap from different CA3 cells, and the ability of attractor networks to operate with diluted connectivity shown in our computational studies prompted by this issue (Treves, 1990; Treves and Rolls, 1991; Rolls, 2012a; Rolls and Webb, 2012).

Crucial issues include how many memories could be stored in this system (to determine whether the autoassociation hypothesis leads to a realistic estimate of the number of memories that the hippocampus could store); whether the whole of a memory could be completed from any part; whether the autoassociation memory can act as a short-term memory, for which the architecture is inherently suited; and whether the system could operate with spatial representations, which are essentially continuous because of the continuous nature of space. These and related issues are considered in the remainder of section CA3 as an Autoassociation or Attractor Memory and in more detail elsewhere (Rolls and Kesner, 2006; Rolls, 2008).

#### Storage capacity

We have performed quantitative analyses of the storage and retrieval processes in the CA3 network (Treves and Rolls, 1991, 1992; Webb et al., 2011; Rolls, 2012a; Rolls and Webb, 2012). We have extended previous formal models of autoassociative memory (see Amit, 1989) by analyzing a network with graded response units, so as to represent more realistically the continuously variable rates at which neurons fire, and with incomplete connectivity (Treves, 1990; Treves and Rolls, 1991; Rolls et al., 1997b; Webb et al., 2011). We have found that in general the maximum number *p*_max_ of firing patterns that can be (individually) retrieved is proportional to the number *C*^RC^ of (associatively) modifiable recurrent collateral synapses on to each neuron, by a factor that increases roughly with the inverse of the sparseness *a* of the neuronal representation. [Each memory is precisely defined in the theory: it is a set of firing rates of the population of neurons (which represent a memory) that can be stored and later retrieved, with retrieval being possible from a fraction of the originally stored set of neuronal firing rates.] The neuronal population sparseness *a* of the representation can be measured by extending the binary notion of the proportion of neurons that are firing to any one stimulus or event as,

(2) $$a={\left(\sum_{i\;=\;1,n}r_{i}/N\right)}^{\text{​​}2}\text{​​}/\text{​​}\sum_{i=1,n}\left(r_{i}^{2}/N\right)$$

where *r*_*i*_ is the firing rate of the *i*'th neuron in the set of *N* neurons. The sparseness ranges from 1/*N*, when only one of the neurons responds to a particular stimulus (a local or grandmother cell representation), to a value of 1.0, attained when all the neurons are responding to a given stimulus. Approximately,

(3) $$p_{\max}≅\frac{C^{\text{RC}}}{\left[a\ln(1/a)\right]}k$$

where *k* is a factor that depends weakly on the detailed structure of the rate distribution, on the connectivity pattern, etc., but is roughly in the order of 0.2-0.3 (Treves and Rolls, 1991). For example, for *C*^RC^ = 12.000 and *a* = 0.02, *p*_max_ is calculated to be ~36,000. This analysis emphasizes the utility of having a sparse representation in the hippocampus, for this enables many different memories to be stored. [The sparseness *a* in this equation is strictly the population sparseness (Treves and Rolls, 1991; Franco et al., 2007). The population sparseness *a*^*p*^ would be measured by measuring the distribution of firing rates of all neurons to a single stimulus at a single time. The single neuron sparseness or selectivity *a*^*s*^ would be measured by the distribution of firing rates to a set of stimuli, which would take a long time. The selectivity or sparseness *a*^*s*^ of a single neuron measured across a set of stimuli often takes a similar value to the population sparseness *a*^*p*^ in the brain, and does so if the tuning profiles of the neurons to the set of stimuli are uncorrelated (Franco et al., 2007). These concepts are elucidated by Franco et al. (2007)]. (I note that the sparseness estimates obtained by measuring early gene changes, which are effectively population sparsenesses, would be expected to depend greatly on the range of environments or stimuli in which these were measured. If the environment was restricted to one stimulus, this would reflect the population sparseness. If the environment was changing, the measure from early gene changes would be rather undefined, as all the populations of neurons activated in an undefined number of testing situations would be likely to be activated.)

In order for most associative networks to store information efficiently, heterosynaptic Long-Term Depression (as well as LTP) is required (Rolls and Treves, 1990, 1998; Treves and Rolls, 1991; Fazeli and Collingridge, 1996; Rolls and Deco, 2002; Rolls, 2008). Simulations that are fully consistent with the analytic theory are provided by Rolls (1995, 2012a), Simmen et al. (1996) and Rolls et al. (1997b).

A number of points that arise, including measurement of the total amount of information (in bits per synapse) that can be retrieved from the network, the computational definition of a memory, the computational sense in which CA3 is an attractor network, and the possible computational utility of memory reconsolidation, are treated elsewhere (Rolls and Kesner, 2006; Rolls, 2008). Here I note that given that the memory capacity of the hippocampal CA3 system is limited, it is necessary to have some form of forgetting in this store, or other mechanism to ensure that its capacity is not exceeded. (Exceeding the capacity can lead to a loss of much of the information retrievable from the network.) Heterosynaptic LTD could help this *forgetting*, by enabling new memories to overwrite old memories (Rolls, 1996a, 2008). The limited capacity of the CA3 system does also provide one of the arguments that some transfer of information from the hippocampus to neocortical memory stores may be useful (see Treves and Rolls, 1994). Given its limited capacity, the hippocampus might be a useful store for only a limited period, which might be in the order of days, weeks, or months. This period may well depend on the acquisition rate of new episodic memories. If the animal were in a constant and limited environment, then as new information is not being added to the hippocampus, the representations in the hippocampus would remain stable and persistent. These hypotheses have clear experimental implications, both for recordings from single neurons and for the gradient of retrograde amnesia, both of which might be expected to depend on whether the environment is stable or frequently changing. They show that the conditions under which a gradient of retrograde amnesia might be demonstrable would be when large numbers of new memories are being acquired, not when only a few memories (few in the case of the hippocampus being less than a few hundred) are being learned.

It has been suggested that the feedforward connectivity from the entorhinal cortex via the pp to the CA3 neurons may act as a feedforward pattern association network that is more important than the CA3-CA3 recurrent collateral autoassociation system (Cheng, 2013). The quantitative properties of pattern association networks are described elsewhere (Rolls and Treves, 1990, 1998; Rolls, 2008). The analyses described in these sources shows that the capacity of pattern association networks (the maximum number of memories that can be stored and retrieved, here denoted by *p*_max_) is approximately,

(4) $$p_{\max}\approx \frac{C^{\text{PA}}}{\left[a_{0}\ln(1/a_{0})\right]}$$

where *C*^PA^ is the number of feedforward associatively modifiable connections per neuron, and *a*_0_ is the sparseness of the representation in the output neurons of the pattern associator (Rolls, 2008). Given that there are fewer feedforward (pp) synaptic connections onto CA3 neurons (3600) than recurrent synaptic connections between CA3 neurons (12,000 in the rat; see Figure 2), then the capacity of the feedforward system would be considerably smaller than that of the recurrent collateral CA3-CA3 system. (It is noted that the *a*_0_ of Equation 4 would be the same number as the *a* of Equation (3), as that is just the sparseness of the firing of the population of CA3 neurons. The number of pp synapses is sufficiently large that it can act as a retrieval cue for even an incomplete pattern so that the CA3-CA3 connections can then complete the retrieval, given that the recall signal for the pp pattern associator is proportional to the square root of the number of pp synapses, as shown by Equation 17 of Treves and Rolls (1992). The feedforward hypothesis (Cheng, 2013) thus has a strong argument against it of storage capacity, which would be much less (approximately 3600/12,000) than that of the CA3-CA3 recurrent collateral system operating as an autoassociation memory. Another disadvantage of the feedforward hypothesis is that the attractor properties of the CA3-CA3 connections would be lost, and these potentially contribute to holding one or more items simultaneously active in short-term memory (Rolls, 2008; Rolls et al., 2013), and providing a basis for temporal order memory as described in section Temporal Order Memory in the Hippocampus, and Episodic Memory. Another disadvantage is that we have been able to show (Treves and Rolls, 1992) that an input of the pp type, alone, is unable to direct efficient information storage. Such an input is too weak, it turns out, to drive the firing of the cells, as the “dynamics” of the network is dominated by the randomizing effect of the recurrent collaterals. Another disadvantage of the feedforward hypothesis is that a pattern associator may not with an incomplete cue be able to recall the exact pattern that was stored, whereas an attractor network has the property that it can fall into an attractor basin that can reflect perfect retrieval of the memory (Rolls and Treves, 1998; Rolls, 2008).

#### Recall and completion

A fundamental property of the autoassociation model of the CA3 recurrent collateral network is that the recall can be symmetric, that is, the whole of the memory can be retrieved and completed from any part (Rolls and Treves, 1998; Rolls and Kesner, 2006; Rolls, 2008). For example, in an object-place autoassociation memory, an object could be recalled from a place retrieval cue, and vice versa. In a test of this, Day et al. (2003) trained rats in a study phase to learn in one trial an association between two flavors of food and two spatial locations. During a recall test phase they were presented with a flavor which served as a cue for the selection of the correct location. They found that injections of an NMDA receptor blocker (AP5) or AMPA/kainate receptor blocker (CNQX) to the dorsal hippocampus prior to the study phase impaired encoding, but injections of AP5 prior to the test phase did not impair the place recall, whereas injections of CNQX did impair the place recall. The interpretation is that somewhere in the hippocampus NMDA receptors are necessary for learning one-trial odor-place associations, and that recall can be performed without further involvement of NMDA receptors.

Evidence that the CA3 system is not necessarily required during recall in a reference memory spatial task, such as the water maze spatial navigation for a single spatial location task, is that CA3 lesioned rats are not impaired during recall of a previously learned water maze task (Brun et al., 2002; Florian and Roullet, 2004). However, if completion from an incomplete cue is needed, then CA3 NMDA receptors are necessary (presumably to ensure satisfactory CA3-CA3 learning) even in a reference memory task (Nakazawa et al., 2002; Gold and Kesner, 2005). Thus, the CA3 system appears to be especially needed in rapid, one-trial object-place recall, and when completion from an incomplete cue is required (see further section Tests of the theory). Especially important though in assessing the implications of all such tests is that the theory sets out how the system operates when large numbers of memories, in the order of thousands, are to be stored and retrieved, and this is difficult to test adequately in behavioral experiments. Effects found when the storage and retrieval of just a few memories are tested may not reflect well the operation of the system when it is heavily loaded, as it is expected to be when operating in the natural environment.

#### Continuous, spatial, patterns, and CA3 representations

The fact that spatial patterns, which imply continuous representations of space, are represented in the hippocampus has led to the application of continuous attractor models to help understand hippocampal function. This has been necessary, because space is inherently continuous, because the firing of place and spatial view cells is approximately Gaussian as a function of the distance away from the preferred spatial location, because these cells have spatially overlapping fields, and because the theory is that these cells in CA3 are connected by Hebb-modifiable synapses. This specification would inherently lead the system to operate as a continuous attractor network. Continuous attractor network models have been studied by Amari (1977), Zhang (1996), Taylor (1999), Samsonovich and McNaughton (1997), Battaglia and Treves (1998), Stringer et al. (2002a,b, 2004), Stringer and Rolls (2002) and Rolls and Stringer (2005) (see Rolls and Deco, 2002; Rolls, 2008), and are described briefly next.

A “Continuous Attractor” neural network (CANN) can maintain the firing of its neurons to represent any location along a continuous physical dimension such as spatial view, spatial position, head direction, etc. It uses excitatory recurrent collateral connections between the neurons (as are present in CA3) to reflect the distance between the neurons in the state space of the animal (e.g., place or head direction). These networks can maintain the bubble or packet of neural activity constant for long periods wherever it is started to represent the current state (head direction, position, etc.) of the animal, and are likely to be involved in many aspects of spatial processing and memory, including spatial vision. Global inhibition is used to keep the number of neurons in a bubble or packet of actively firing neurons relatively constant, and to help to ensure that there is only one activity packet.

**Figure 4:**
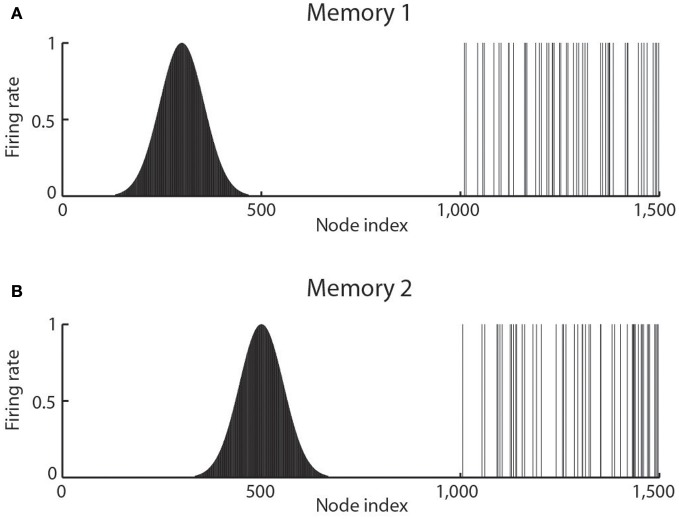
**The types of firing patterns stored in continuous attractor networks are illustrated for the patterns present on neurons 1–1000 for Memory 1 (when the firing is that produced when the spatial state represented is that for location 300), and for Memory 2 (when the firing is that produced when the spatial state represented is that for location 500)**. The continuous nature of the spatial representation results from the fact that each neuron has a Gaussian firing rate that peaks at its optimal location. This particular mixed network also contains discrete representations that consist of discrete subsets of active binary firing rate neurons in the range 1001–1500. The firing of these latter neurons can be thought of as representing the discrete events that occur at the location. Continuous attractor networks by definition contain only continuous representations, but this particular network can store mixed continuous and discrete representations, and is illustrated to show the difference of the firing patterns normally stored in separate continuous attractor and discrete attractor networks. For this particular mixed network, during learning, Memory 1 is stored in the synaptic weights, then Memory 2, etc., and each memory contains part that is continuously distributed to represent physical space, and part that represents a discrete event or object.

Continuous attractor networks can be thought of as very similar to autoassociation or discrete attractor networks (Rolls, 2008), and have the same architecture, as illustrated in Figure 3. The main difference is that the patterns stored in a CANN are continuous patterns, with each neuron having broadly tuned firing which decreases with for example a Gaussian function as the distance from the optimal firing location of the cell is varied, and with different neurons having tuning that overlaps throughout the space. Such tuning is illustrated in Figure 4. For comparison, autoassociation networks normally have discrete (separate) patterns (each pattern implemented by the firing of a particular subset of the neurons), with no continuous distribution of the patterns throughout the space (see Figure 4). A consequent difference is that the CANN can maintain its firing at any location in the trained continuous space, whereas a discrete attractor or autoassociation network moves its population of active neurons toward one of the previously learned attractor states, and thus implements the recall of a particular previously learned pattern from an incomplete or noisy (distorted) version of one of the previously learned patterns.

Space is continuous, and object representations are discrete. If these representations are to be combined in for example an object-place memory, then we need to understand the operation of networks that combine these representations. Rolls et al. (2002) have shown that attractor networks can store both continuous patterns and discrete patterns (as illustrated in Figure 4), and can thus be used to store for example the location in (continuous, physical) space (e.g., the place “out there” in a room represented by spatial view cells) where an object (a discrete item) is present. We showed this by storing associated continuous and discrete representations in the same single attractor network, and then showing that the representation in the continuous space could be retrieved by the discrete object that was associated with that spatial position; and that the representation of the discrete object could be retrieved by providing the position in the continuous representation of space.

If spatial representations are stored in the hippocampus, the important issue arises in terms of understanding memories that include a spatial component or context of how many such spatial representations could be stored in a continuous attractor network. The very interesting result is that because there are in general low correlations between the representations of places in different maps or charts (where each map or chart might be of one room or locale), very many different maps of charts can be simultaneously stored in a continuous attractor network (Battaglia and Treves, 1998).

We have considered how spatial representations could be stored in continuous attractor networks, and how the activity can be maintained at any location in the state space in a form of short-term memory when the external (e.g., visual) input is removed. However, a property of some spatial representations is that they can be updated by self-motion, idiothetic, input, and mechanisms have been proposed for how this could be achieved (Samsonovich and McNaughton, 1997; Stringer et al., 2002a,b, 2005; Rolls and Stringer, 2005; Stringer and Rolls, 2006; Walters et al., 2013), including in the entorhinal cortex grid cell system (Kropff and Treves, 2008; Giocomo et al., 2011; Zilli, 2012). The ways in which path integration could be implemented in recurrent networks such as the CA3 system in the hippocampus or in related systems are described elsewhere (Samsonovich and McNaughton, 1997; Stringer et al., 2002a,b; McNaughton et al., 2006), and have been applied to primate spatial view cells by Rolls and colleagues (Stringer et al., 2004, 2005; Rolls and Stringer, 2005). Cognitive maps (O'Keefe and Nadel, 1978) can be understood by the operations of these attractor networks, and how they are updated by learning and by self-motion (Rolls, 2008). It has been argued that the bumpiness of the CA3 representation of space is more consistent with episodic memory storage, as argued in this paper, than with spatial path integration using the CA3 system as a continuous attractor network implementing path integration (Cerasti and Treves, 2013; Stella et al., 2013).

#### Mossy fiber inputs to the CA3 cells

We hypothesize that the MF inputs force efficient information storage by virtue of their strong and sparse influence on the CA3 cell firing rates (Rolls, 1987, 1989b,c; Treves and Rolls, 1992). [The strong effects likely to be mediated by the MFs were also emphasized by McNaughton and Morris (1987) and McNaughton and Nadel (1990)]. We (Rolls and Treves) (Rolls, 1987, 1989b,c, 1990b; Treves and Rolls, 1992; Rolls and Treves, 1998; Rolls, 2008) hypothesize that the MF input appears to be particularly appropriate in several ways. First, the fact that MF synapses are large and located very close to the soma makes them relatively powerful in activating the postsynaptic cell. Second, the firing activity of dentate granule cells appears to be very sparse (Jung and McNaughton, 1993; Leutgeb et al., 2007) and this, together with the small number of connections on each CA3 cell, produces a sparse signal, which can then be transformed into sparse firing activity in CA3 by a threshold effect. The hypothesis is that the MF sparse connectivity solution performs the appropriate function to enable learning to operate correctly in CA3 (Treves and Rolls, 1992; Cerasti and Treves, 2010). The pp input would, the quantitative analysis shows, not produce a pattern of firing in CA3 that contains sufficient information for learning (Treves and Rolls, 1992) (see further section Perforant Path Inputs to CA3 Cells).

The particular property of the small number of MF connections onto a CA3 cell, ~46 (see Figure 2), is that this has a **randomizing effect** on the representations set up in CA3, so that they are as different as possible from each other (Rolls, 1989c,b; Treves and Rolls, 1992; Rolls and Treves, 1998; Rolls and Kesner, 2006; Rolls, 2008). (This means for example that place cells in a given environment are well-separated to cover the whole space.) The result is that any one event or episode will set up a representation that is very different from other events or episodes, because the set of CA3 neurons activated for each event is random. This is then the optimal situation for the CA3 recurrent collateral effect to operate, for it can then associate together the random set of neurons that are active for a particular event (for example an object in a particular place), and later recall the whole set from any part. It is because the representations in CA3 are unstructured, or random, in this way that large numbers of memories can be stored in the CA3 autoassociation system, and that interference between the different memories is kept as low as possible, in that they are maximally different from each other (Hopfield, 1982; Treves and Rolls, 1991; Rolls and Treves, 1998; Rolls, 2008).

The requirement for a small number of MF connections onto each CA3 neuron applies not only to discrete (Treves and Rolls, 1992) but also to spatial representations, and some learning in these connections, whether associative or not, can help to select out the small number of MFs that may be active at any one time to select a set of random neurons in the CA3 (Cerasti and Treves, 2010). Any learning may help by reducing the accuracy required for a particular number of MF connections to be specified genetically onto each CA3 neuron. The optimal number of MFs for the best information transfer from dentate granule cells to CA3 cells is in the order of 35–50 (Treves and Rolls, 1992; Cerasti and Treves, 2010). The MFs also make connections useful for feedforward inhibition (Acsady et al., 1998).

On the basis of these and other points, we predicted that the MFs may be necessary for new learning in the hippocampus, but may not be necessary for the recall of existing memories from the hippocampus (Treves and Rolls, 1992; Rolls and Treves, 1998; Rolls, 2008). Experimental evidence consistent with this prediction about the role of the MFs in learning has been found in rats with disruption of the dentate granule cells (Lassalle et al., 2000) (section Tests of the theory).

We (Rolls and Kesner, 2006) have hypothesized that non-associative plasticity of MFs (see Brown et al., 1989, 1990) might have a useful effect in enhancing the signal-to-noise ratio, in that a consistently firing MF would produce non-linearly amplified currents in the postsynaptic cell, which would not happen with an occasionally firing fiber (Treves and Rolls, 1992). This plasticity, and also learning in the dentate, would also have the effect that similar fragments of each episode (e.g., the same environmental location) recurring on subsequent occasions would be more likely to activate the same population of CA3 cells, which would have potential advantages in terms of economy of use of the CA3 cells in different memories, and in making some link between different episodic memories with a common feature, such as the same location in space. Consistent with this, dentate neurons that fire repeatedly are more effective in activating CA3 neurons (Henze et al., 2002).

As acetylcholine turns down the efficacy of the recurrent collateral synapses between CA3 neurons (Hasselmo et al., 1995; Giocomo and Hasselmo, 2007), then cholinergic activation also might help to allow external inputs rather than the internal recurrent collateral inputs to dominate the firing of the CA3 neurons during learning, as the current theory proposes. If cholinergic activation at the same time facilitated LTP in the recurrent collaterals (as it appears to in the neocortex), then cholinergic activation could have a useful double role in facilitating new learning at times of behavioral activation (Hasselmo et al., 1995; Giocomo and Hasselmo, 2007), when presumably it may be particularly relevant to allocate some of the limited memory capacity to new memories.

#### Perforant path inputs to CA3 cells

By calculating the amount of information that would end up being carried by a CA3 firing pattern produced solely by the pp input and by the effect of the recurrent connections, we have been able to show (Treves and Rolls, 1992) that an input of the pp type, alone, is unable to direct efficient information storage. Such an input is too weak, it turns out, to drive the firing of the cells, as the “dynamics” of the network is dominated by the randomizing effect of the recurrent collaterals. On the other hand, an autoassociative memory network needs afferent inputs to apply the retrieval cue to the network. We have shown (Treves and Rolls, 1992) that the pp system is likely to be the one involved in relaying the cues that initiate retrieval in CA3. The concept is that to initiate retrieval, a numerically large input (the pp system, see Figure 2) is useful so that even a partial cue is sufficient [see Equationn 17 of Treves and Rolls (1992)]; and that the retrieval cue need not be very strong, as the recurrent collaterals (in CA3) then take over in the retrieval process to produce good recall (Treves and Rolls, 1992; Rolls, 2008). In contrast, during storage, strong signals, in the order of mV for each synaptic connection, are provided by the MF inputs to dominate the recurrent collateral activations, so that the new pattern of CA3 cell firing can be stored in the CA3 recurrent collateral connections (Treves and Rolls, 1992; Rolls, 2008).

#### Why is the CA3 recurrent collateral connectivity diluted?

Figure 2 shows that in the rat, there are ~300,000 CA3 neurons, but only 12,000 recurrent collateral synapses per neuron. The dilution of the connectivity is thus 12,000/300,000 = 0.04. The connectivity is thus not complete, and complete connectivity in an autoassociation network would make it simple, for the connectivity between the neurons would then be symmetric (i.e., the connection strength from any one neuron to another is matched by a connection of the same strength in the opposite direction), and this guarantees energy minima for the basins of attraction that will be stable, and a memory capacity than can be calculated (Hopfield, 1982). We have shown how this attractor type of network can be extended to have similar properties with diluted connectivity, and also with sparse representations with graded firing rates (Rolls and Treves, 1990; Treves, 1990, 1991; Treves and Rolls, 1991).

However, the question has recently been asked about whether there are any advantages to diluted autoassociation or attractor networks compared to fully connected attractor networks (Rolls, 2012a). One biological property that may be a limiting factor is the number of synaptic connections per neuron, which is 12,000 in the CA3-CA3 network just for the recurrent collaterals (see Figure 2). The number may be higher in humans, allowing more memories to be stored in the hippocampus than order 12,000. I note that the storage of large number of memories may be facilitated in humans because the left and right hippocampus appear to be much less connected between the two hemispheres than in the rat, which effectively has a single hippocampus (Rolls, 2008). In humans, with effectively two separate CA3 networks, one on each side of the brain, the memory storage capacity may be doubled, as the capacity is set by the number of recurrent collaterals per neuron in each attractor network (Equation 3). In humans, the right hippocampus may be devoted to episodic memories with spatial and visual components, whereas the left hippocampus may be devoted to memories with verbal/linguistic components, i.e., in which words may be part of the episode (e.g., who said what to whom and when; Barkas et al., 2010; Bonelli et al., 2010; Sidhu et al., 2013).

The answer that has been suggested to why the connectivity of the CA3 autoassociation network is diluted (and why neocortical recurrent networks are also diluted), is that this may help to reduce the probability of having two or more synapses between any pair of randomly connected neurons within the network, which it has been shown greatly impairs the number of memories that can be stored in an attractor network, because of the distortion that this produces in the energy landscape (Rolls, 2012a). In more detail, the hypothesis proposed is that the diluted connectivity allows biological processes that set up synaptic connections between neurons to arrange for there to be only very rarely more than one synaptic connection between any pair of neurons. If probabilistically there were more than one connection between any two neurons, it was shown by simulation of an autoassociation attractor network that such connections would dominate the attractor states into which the network could enter and be stable, thus strongly reducing the memory capacity of the network (the number of memories that can be stored and correctly retrieved), below the normal large capacity for diluted connectivity. Diluted connectivity between neurons in the cortex thus has an important role in allowing high capacity of memory networks in the cortex, and helping to ensure that the critical capacity is not reached at which overloading occurs leading to an impairment in the ability to retrieve any memories from the network (Rolls, 2012a). The diluted connectivity is thus seen as an adaptation that simplifies the genetic specification of the wiring of the brain, by enabling just two attributes of the connectivity to be specified (e.g., from a CA3 to another CA3 neuron chosen at random to specify the CA3 to CA3 recurrent collateral connectivity), rather than which particular neuron should connect to which other particular neuron (Rolls and Stringer, 2000; Rolls, 2012a). Consistent with this hypothesis, there are NMDA receptors with the genetic specification that they are NMDA receptors on neurons of a particular type, CA3 neurons (as shown by the evidence from CA3-specific vs. CA1-specific NMDA receptor knockouts; Rondi-Reig et al., 2001; Nakazawa et al., 2002, 2003, 2004). A consequence is that the vector of output neuronal firing in the CA3 regions, i.e., the number of CA3 neurons, is quite large (300,000 neurons in the rat). The large number of elements in this vector may have consequences for the noise in the system, as we will see below.

The dilution of the CA3-CA3 recurrent collateral connectivity at 0.04 may be greater dilution than that in a local neocortical area, which is in the order of 0.1 (Rolls, 2008, 2012a). This is consistent with the hypothesis that the storage capacity of the CA3 system is at a premium, and so the dilution is kept to a low value (i.e., great dilution), as then there is lower distortion of the basins of attraction and hence the memory capacity is maximized (Rolls, 2012a).

#### Noise and stability produced by the diluted connectivity and the graded firing rates in the CA3-CA3 attractor network

Many processes in the brain are influenced by the noise or variability of neuronal spike firing (Faisal et al., 2008; Rolls and Deco, 2010; Deco et al., 2013). The action potentials are generated in a way that frequently approximates a Poisson process, in which the spikes for a given mean firing rate occur at times that are essentially random (apart from a small effect of the refractory period), with a coefficient of variation (CV) of the interspike interval distribution near 1.0 (Rolls and Deco, 2010). The sources of the noise include quantal transmitter release, and noise in ion channel openings (Faisal et al., 2008). The membrane potential is often held close to the firing threshold, and then small changes in the inputs and the noise in the neuronal operations cause spikes to be emitted at almost random times for a given mean firing rate. Spiking neuronal networks with balanced inhibition and excitation currents and associatively modified recurrent synaptic connections can be shown to possess a stable attractor state where neuron spiking is approximately Poisson too (Amit and Brunel, 1997; Miller and Wang, 2006). The noise caused by the variability of individual neuron spiking which then affects other neurons in the network can play an important role in the function of such recurrent attractor networks, by causing for example an otherwise stable network to jump into a decision state (Wang, 2002; Deco and Rolls, 2006; Rolls and Deco, 2010). Attractor networks with this type of spiking-related noise are used in the brain for memory recall, and for decision-making, which in terms of the neural mechanism are effectively the same process (Rolls, 2008). Noise in attractor networks is useful for memory and decision-making, for it makes them non-deterministic, and this contributes to new solutions to problems, and indeed to creativity (Rolls and Deco, 2010).

To investigate the extent to which this diluted connectivity affects the dynamics of attractor networks in the cerebral cortex (which includes the hippocampus), we simulated an integrate-and-fire attractor network taking decisions between competing inputs with diluted connectivity of 0.25 or 0.1 but the same number of synaptic connections per neuron for the recurrent collateral synapses within an attractor population as for full connectivity (Rolls and Webb, 2012). The results indicated that there was less spiking-related noise with the diluted connectivity in that the stability of the network when in the spontaneous state of firing increased, and the accuracy of the correct decisions increased. The decision times were a little slower with diluted than with complete connectivity. Given that the capacity of the network is set by the number of recurrent collateral synaptic connections per neuron, on which there is a biological limit, the findings indicate that the stability of cortical networks, and the accuracy of their correct decisions or memory recall operations, can be increased by utilizing diluted connectivity and correspondingly increasing the number of neurons in the network (which may help to smooth the noise), with little impact on the speed of processing of the cortex. Thus, diluted connectivity can decrease cortical spiking-related noise, and thus enhance the reliability of memory recall (Rolls and Webb, 2012).

Representations in the neocortex and in the hippocampus are often distributed with graded firing rates in the neuronal populations (Rolls and Treves, 2011). The firing rate probability distribution of each neuron to a set of stimuli is often exponential or gamma (Rolls and Treves, 2011). These graded firing rate distributed representations are present in the hippocampus, both for place cells in rodents and for spatial view cells in the primate (O'Keefe, 1979; McNaughton et al., 1983; O'Keefe and Speakman, 1987; Rolls et al., 1997a, 1998; Robertson et al., 1998; Georges-François et al., 1999; Rolls, 2008; Rolls and Treves, 2011). In processes in the brain such as memory recall in the hippocampus or decision-making in the cortex that are influenced by the noise produced by the close to random spike timings of each neuron for a given mean rate, the noise with this graded type of representation may be larger than with the binary firing rate distribution that is usually investigated. In integrate-and-fire simulations of an attractor decision-making network, we showed that the noise is indeed greater for a given sparseness of the representation for graded, exponential, than for binary firing rate distributions (Webb et al., 2011). The greater noise was measured by faster escaping times from the spontaneous firing rate state when the decision cues are applied, and this corresponds to faster decision or reaction times. The greater noise was also evident as less stability of the spontaneous firing state before the decision cues are applied. The implication is that spiking-related noise will continue to be a factor that influences processes such as decision-making, signal detection, short-term memory, and memory recall (including in the CA3 network) even with the quite large networks found in the cerebral cortex. In these networks there are several thousand recurrent collateral synapses onto each neuron. The greater noise with graded firing rate distributions has the advantage that it can increase the speed of operation of cortical circuitry (Webb et al., 2011).

### Dentate granule cells

The theory is that the dentate granule cell stage of hippocampal processing which precedes the CA3 stage acts as a competitive network in a number of ways to produce during learning the sparse yet efficient (i.e., non-redundant) representation in CA3 neurons that is required for the autoassociation implemented by CA3 to perform well (Rolls, 1989b,c, 1990b; Treves and Rolls, 1992; Rolls and Kesner, 2006; Rolls et al., 2006). An important property for episodic memory is that the dentate by acting in this way would perform pattern separation (or orthogonalization) (Rolls, 1989b; Treves and Rolls, 1992; Rolls and Kesner, 2006; Rolls et al., 2006), enabling the hippocampus to store different memories of even similar events, and this prediction has been confirmed (Gilbert et al., 2001; Rolls and Kesner, 2006; Leutgeb and Leutgeb, 2007; McHugh et al., 2007; Goodrich-Hunsaker et al., 2008; Rolls, 2008; Kesner et al., 2012) (section Tests of the theory).

As just described, the dentate granule cells could be important in helping to build and prepare spatial representations for the CA3 network. The actual representation of space in the primate hippocampus includes a representation of spatial view (Rolls and Xiang, 2006), whereas in the rat hippocampus it is of the place where the rat is. The representation in the rat may be related to the fact that with a much less developed visual system than the primate, the rat's representation of space may be defined more by the olfactory and tactile as well as distant visual cues present, and may thus tend to reflect the place where the rat is. However, the spatial representations in the rat and primate could arise from essentially the same computational process as follows (Rolls, 1999; De Araujo et al., 2001). The starting assumption is that in both the rat and the primate, the dentate granule cells (and the CA3 and CA1 pyramidal cells) respond to combinations of the inputs received. In the case of the primate, a combination of visual features in the environment will, because of the fovea providing high spatial resolution over a typical viewing angle of perhaps 10–20°, result in the formation of a spatial view cell, the effective trigger for which will thus be a combination of visual features within a relatively small part of space. In contrast, in the rat, given the very extensive visual field subtended by the rodent retina, which may extend over 180–270°, a combination of visual features formed over such a wide visual angle would effectively define a position in space that is a place (De Araujo et al., 2001).

The entorhinal cortex contains grid cells, which have high firing in the rat in a two-dimensional spatial grid as a rat traverses an environment, with larger grid spacings in the ventral entorhinal cortex (Fyhn et al., 2004; Hafting et al., 2005). This may be a system optimized for path integration (McNaughton et al., 2006) which may self-organize during locomotion with longer time constants producing more widely spaced grids in the ventral entorhinal cortex (Kropff and Treves, 2008). How are the grid cell representations, which would not be suitable for association of an object or reward with a place to form an episodic memory, transformed into a place representation that would be appropriate for this type of episodic memory? I have proposed that this could be implemented by a competitive network (Rolls, 2008) in the DG which operates to form place cells, implemented by each dentate granule cell learning to respond to particular combinations of entorhinal cortex cells firing, where each combination effectively specifies a place, and this has been shown to be feasible computationally (Rolls et al., 2006). The sparse representations in the DG, implemented by the mutual inhibition through inhibitory interneurons and competitive learning, help to implement this “pattern separation” effect (Rolls, 1989b,c; Rolls and Treves, 1998; Rolls, 2008).

In primates, there is now evidence that there is a grid-cell like representation in the entorhinal cortex, with neurons having grid-like firing as the monkey moves the eyes across a spatial scene (Killian et al., 2012). Similar competitive learning processes may transform these entorhinal cortex “spatial view grid cells” into hippocampal spatial view cells, and may help with the idiothetic (produced in this case by movements of the eyes) update of spatial view cells (Robertson et al., 1998). The presence of spatial view grid cells in the entorhinal cortex of primates (Killian et al., 2012) is of course predicted from the presence of spatial view cells in the primate CA3 and CA1 regions (Rolls et al., 1997a, 1998; Robertson et al., 1998; Georges-François et al., 1999; Rolls and Xiang, 2006; Rolls, 2008).

In primates, spatial view cells represent a scene view allocentrically, as described in section Systems-Level Neurophysiology of the Primate Hippocampus. How could such spatial view representations be formed, in which the relative spatial position of features in a scene is encoded? I have proposed that this involves competitive learning analogous to that used to form place cells in rats, but in primates operating on the representations of objects that reach the hippocampus from the inferior temporal visual cortex (Rolls et al., 2008b). We have shown that in complex natural scenes the receptive fields of inferior temporal cortex neurons become reduced in size and asymmetric with respect to the fovea (Aggelopoulos and Rolls, 2005; Rolls, 2009), and Rolls et al. (2008b) have demonstrated in a unifying computational approach that competitive network processes operating in areas such as the parahippocampal cortex, the entorhinal cortex, and/or the dentate granule cells could form unique views of scenes by forming a sparse representation of these object or feature tuned inferior temporal cortex ventral visual stream representations which have some spatial asymmetry.

### CA1 cells

The CA3 cells connect to the CA1 cells by the Schaeffer collateral synapses. The associative modifiability in this connection helps the full information present in CA3 to be retrieved in the CA1 neurons (Treves and Rolls, 1994; Rolls, 1995; Treves, 1995; Schultz and Rolls, 1999). Part of the hypothesis is that the separate sub-parts of an episodic memory, which must be represented separately in CA3 to allow for completion, can be combined together by competitive learning in CA1 to produce an efficient retrieval cue for the recall via the backprojection pathways to the neocortex of memories stored in the neocortex (Rolls, 1989a,b; Treves and Rolls, 1994; Rolls, 1995, 1996b). Associative recall in the CA3 to CA1 feedforward connections is a prominent property (Rolls, 1995; Schultz et al., 2000).

### Backprojections to the neocortex, and memory recall

The need for information to be retrieved from the hippocampus to affect other brain areas was noted in the Introduction. The way in which this could be implemented via backprojections to the neocortex is considered elsewhere (Treves and Rolls, 1994; Rolls, 1995, 1996b, 2008, 2010b) (see Figure 1).

### Temporal order memory in the hippocampus, and episodic memory

There has for some time been evidence that the hippocampus plays a role in temporal order memory, even when there is no spatial component (Kesner et al., 2002; Rolls and Kesner, 2006; Hoge and Kesner, 2007). In humans, the hippocampus becomes activated when the temporal order of events is being processed (Lehn et al., 2009). If this is a function that can be implemented in the hippocampus, I now propose that this could be very important for understanding episodic memory, which often may comprise a temporal sequence of events. However, until recently it has not been clear how temporal order memory could be implemented in the hippocampus, or, for that matter, in other brain structures. However, the outline of a theory of temporal order memory, and how it could be implemented in the hippocampus, has now been developed (Rolls and Deco, 2010), as follows.

The approach is based on recent neurophysiological evidence of MacDonald et al. (2011) showing that neurons in the rat hippocampus have firing rates that reflect which temporal part of the task is current. In particular, a sequence of different neurons is activated at successive times during a time delay period. The tasks used included an object-odor paired associate non-spatial task with a 10 s delay period between the visual stimulus and the odor. The new evidence also shows that a large proportion of hippocampal neurons fire in relation to individual events in a sequence being remembered (e.g., a visual object or odor), and some to combinations of the event and the time in the delay period (MacDonald et al., 2011).

These interesting neurophysiological findings indicate that rate encoding is being used to encode time, that is, the firing rates of different neurons are high at different times within a trial, delay period, etc. (Rolls and Deco, 2010; MacDonald et al., 2011). This provides the foundation for a new computational theory of temporal order memory within the hippocampus (and also the prefrontal cortex) which I outline next, and which utilizes the slow transitions from one attractor to another which are a feature that arises at least in some networks in the brain due to the noise-influenced transitions from one state to another.

First, because some neurons fire at different times in a trial of a temporal order memory task or delay task, the time in a trial at which an object (e.g., a visual stimulus or odor) was presented could become encoded in the hippocampus by an association implemented in the CA3 recurrent collaterals between the neurons that represent the object [already known to be present in the hippocampus for tasks for which the hippocampus is required (Rolls et al., 2005; Rolls and Xiang, 2006)] and the “time encoding” neurons in the hippocampus (MacDonald et al., 2011). This would allow associations for the time at which the object was present to be formed.

Second, these associations would provide the basis for the recall of the object from the time in a trial, or vice versa. The retrieval of object or temporal information from each other would occur in a way that is analogous to that shown for recalling the object from the place, or the place from the object (Rolls et al., 2002), but substituting the details of the properties of the “time encoding” neurons (MacDonald et al., 2011) for what was previously the spatial (place) component. In addition, if the time encoding neurons simply cycled through their normal sequence during recall, this would enable the sequence of objects or events associated with each subset of time encoding neurons to be recalled correctly in the order in which they were presented.

Third, we need a theory of what the origin is of the temporal effect whereby different hippocampal (or potentially prefrontal cortex) neurons fire in different parts of a trial or delay period. The properties of the “time encoding neurons” (Rolls and Deco, 2010; MacDonald et al., 2011) are key here, and we need to understand how they are generated. Are they generated within the hippocampus, or elsewhere, and in any case, what is the mechanism by which different neurons have high firing rates at different times in a trial? The fundamentally new approach to hippocampal function I am taking here is that rate encoding is being used, that is, the firing rates of different neurons are high at different times within a trial (Rolls and Deco, 2010; MacDonald et al., 2011). This is a radically different approach to order encoding than that based on phenomena such a theta and gamma oscillations that has been investigated by Lisman and colleagues (Lisman and Redish, 2009).

We can consider three hypotheses about how the firing of the “time encoding” hippocampal neurons is produced. All utilize slow transitions between attractor states that can be a property of noisy attractor networks. The first hypothesis is that an attractor network with realistic dynamics (modeled at the integrate-and-fire level with a dynamical implementation of the neuronal membrane and synaptic current dynamics, and with synaptic or neuronal adaptation) can implement a sequence memory (Deco and Rolls, 2005). The hypothesis is that there are several different attractors, and that there are weak connections between the different attractors. In the model, adaptation produces effects whereby whatever sequence (order of stimuli) is presented on an individual trial that order can be replayed in the same sequence because as one attractor state dies as a result of the adaptation, the next attractor to emerge from the spontaneous firing because of the spiking-related noise is the one that has been active least recently, as it is the one that is least adapted (Deco and Rolls, 2005). The whole system operates at a rather slow timescale for the transitions between the attractors partly because of the time for the noise to drive the system from one attractor state to another, and the slow time course of the adaptation (Deco and Rolls, 2005; Rolls and Deco, 2010). This implements a type of order memory.

The second hypothesis is analogous, and is also implemented in a recurrently connected system such as the hippocampal CA3 system or local recurrent circuits in the neocortex (Rolls and Deco, 2010). This second theory is that again there are several attractors, but that each attractor is connected by slightly stronger forward than reverse synaptic weights to the next. In previous work, we have shown that with an integrate-and-fire implementation with spiking noise this allows slow transitions from one attractor state to the next (Deco and Rolls, 2003; Deco et al., 2005). During learning of the synaptic weights in the network, adaptation might lead to each “time encoding” population of neurons responding for only a limited period, helping to produce multiple sequentially activated populations of time encoding neurons (Rolls and Deco, 2010; MacDonald et al., 2011). In this scenario, an associative pool of neurons is unlikely to be helpful, and stronger forward that reverse weights between different attractors each consisting of a different population of “time encoding” neurons would be the essence. It will be of interest to investigate whether this system, because of the noise, is limited to transitions between up to perhaps 7 ± 2 different sequential firing rate states with different neuronal subpopulations for each state, and thus provides an account for the limit of the magical number 7 ± 2 on short-term memory and related types of processing (Miller, 1956), and for the recency part of short-term memory in which the items are naturally recalled in the order in which they were presented. This is the most likely model at present of short-term memory and its natural propensity to store and to recall items in the order in which they were received (Rolls and Deco, 2010).

A variation on this implementation that I have proposed would be to have short-term attractor memories with different time constants (for example of adaptation), but all started at the same time (Rolls and Deco, 2010). This could result in some attractors starting early in the sequence and finishing early, and with other attractors starting up a little later, but lasting for much longer in time. The neurons recorded in the rat (MacDonald et al., 2011) are not inconsistent with this possibility. This type of time-encoding representation could also be used to associate with items, to implement an item-order memory.

It is thus suggested that temporal order memory could be implemented in the hippocampus in this way, and could make an important contribution to episodic memory in which several events linked in the correct order might form an episode. The theory shows how items in a particular temporal order could be separated from each other, a property we have referred to as the temporal pattern separation effect (Rolls and Kesner, 2006). The theory of episodic memory described here shows how events and sequences of events could be recalled from the hippocampus to the neocortex, and there a longer-term more semantic representation of a recalled episode, such as what happened on one's fifth birthday, might be stored, and then accessed to describe the episode. For the order to be correctly implemented in the semantic neocortical store, a similar mechanism, involving for example stronger forward than reverse synaptic weights between long-term memory representations in attractors, could build an appropriate long-term order memory (Rolls and Deco, 2010).

The natural implementation of such temporal order memory would be in the hippocampal CA3-CA3 recurrent collateral network. It is therefore somewhat of a puzzle that some of the evidence implicates the CA1 region in temporal order memory (Rolls and Kesner, 2006; Hunsaker et al., 2008; Kesner et al., 2012). This issue remains to be clarified.

## Systems-level neurophysiology of the primate hippocampus

The systems-level neurophysiology of the hippocampus shows what information could be stored or processed by the hippocampus. To understand how the hippocampus works it is not sufficient to state just that it can store information—one needs to know what information. The systems-level neurophysiology of the primate hippocampus has been reviewed by Rolls and Xiang (2006), and a summary is provided here because it provides a perspective relevant to understanding the function of the human hippocampus that is somewhat different from that provided by the properties of place cells in rodents, which have been reviewed elsewhere (McNaughton et al., 1983; O'Keefe, 1984; Muller et al., 1991; Jeffery et al., 2004; Jeffery and Hayman, 2004).

### Spatial view neurons in the primate hippocampus

We have shown that the primate hippocampus contains spatial cells that respond when the monkey looks at a certain part of space, for example at one quadrant of a video monitor while the monkey is performing an object-place memory task in which he must remember where on the monitor he has seen particular images (Rolls et al., 1989; Rolls, 1999). Approximately 9% of the hippocampal neurons have such spatial view fields, and about 2.4% combine information about the position in space with information about the object that is in that position in space (Rolls et al., 1989). The representation of space is for the majority of hippocampal neurons in allocentric not egocentric coordinates (Feigenbaum and Rolls, 1991). These spatial view cells can be recorded while monkeys move themselves round the test environment by walking (or running) on all fours (Rolls et al., 1997a, 1998; Robertson et al., 1998; Georges-François et al., 1999). These hippocampal “spatial view neurons” respond significantly differently for different allocentric spatial views and have information about spatial view in their firing rate, but do not respond differently just on the basis of eye position, head direction, or place. If the view details are obscured by curtains and darkness, then some spatial view neurons (especially those in CA1 and less those in CA3) continue to respond when the monkey looks toward the spatial view field, showing that these neurons can be updated for at least short periods by idiothetic (self-motion) cues including eye position and head direction signals (Rolls et al., 1997b; Robertson et al., 1998).

### Object-place neurons in the primate hippocampus

A fundamental question about the function of the primate including human hippocampus in relation to episodic memory is whether object as well as allocentric spatial information is represented. To investigate this, Rolls et al. (2005) made recordings from single hippocampal formation neurons while macaques performed an object-place memory task that required the monkeys to learn associations between objects, and where they were shown in a room. Some neurons (10%) responded differently to different objects independently of location; other neurons (13%) responded to the spatial view independently of which object was present at the location; and some neurons (12%) responded to a combination of a particular object and the place where it was shown in the room. These results show that there are separate as well as combined representations of objects and their locations in space in the primate hippocampus. This is a property required in an episodic memory system, for which associations between objects and the places where they are seen, are prototypical. The results thus show that a requirement for a human episodic memory system, separate and combined neuronal representations of objects and where they are seen “out there” in the environment, are present in the primate hippocampus (Rolls et al., 2005).

What may be a corresponding finding in rats is that some rat hippocampal neurons respond on the basis of the conjunction of location and odor (Wood et al., 1999). Results consistent with our object-place neurons in primates are that Diamond and colleagues have now shown using the vibrissa somatosensory input for the “object” system that rat hippocampal neurons respond to object-place combinations, objects, or places, and there is even a reward-place association system in rats similar to that in primates described below (Itskov et al., 2011). This brings the evidence from rats closely into line with the evidence from primates of hippocampal neurons useful for object-place episodic associative memory.

Spatial view cells, and object-place cells, are also present in the parahippocampal areas (Rolls et al., 1997a, 1998, 2005; Robertson et al., 1998; Georges-François et al., 1999). There are backprojections from the hippocampus to the entorhinal cortex and thus to parahippocampal areas, and these backprojections could enable the hippocampus to influence the spatial representations found in the entorhinal cortex and parahippocampal gyrus. On the other hand, some of the spatial functions may be provided for in these parahippocampal areas, which will in turn influence the hippocampus. However, it is argued below that the hippocampus may be able to make a special contribution to event or episodic memory, by enabling in the CA3 network with its very widespread recurrent collateral connections an association between any one item with any other item to form an arbitrary association to represent an event.

### Recall-related neurons in the primate hippocampus

**Figure 5:**
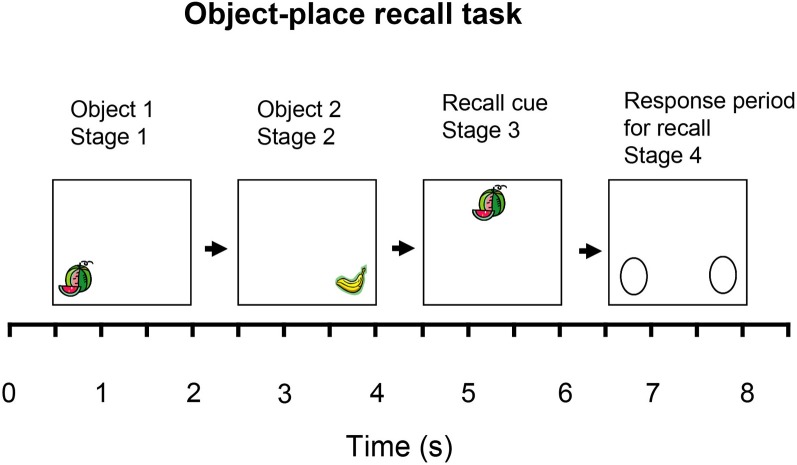
**The object-place recall task**. One-trial is shown. After a 0.5 s tone to indicate the start of a trial, in Stage 1 one of two objects (O1) is shown at one of the places (P1). (The object and the place are chosen randomly on each trial.) To ensure that the monkey sees the stimulus, the monkey can touch the screen at the place to obtain one drop of juice reward by licking. After a 0.5 s delay, in Stage 2, the other of the two objects (O2) is shown at the other place (P2). (One drop of fruit juice was available as in Stage 1.) After a 0.5 s delay, in Stage 3, the recall cue, one of the objects chosen at random, is shown at the top of the screen in the middle. (One drop of fruit juice was available as in stage 1.) After a 0.5 s delay, in Stage 4, the macaque must then recall the place in which the object shown as the recall cue in stage 3 was presented, and must then touch that place on the screen to obtain 4 licks of fruit juice, thus indicating that he has recalled the location correctly. In stage 4 of the trials, the left and right positions (P1 and P2) have no image present, with the two possible locations for a response indicated by identical circles. The task requires the monkey to perform recall of the place from the object, within the period beginning at the presentation of the recall cue at the start of stage 3 and ending when the response is made in stage 4. Reward associations can not be used to solve the task, for the two objects and the two places are not differently associated with reward. Further details are provided by Rolls and Xiang (2006).

It has now been possible to investigate directly, neurophysiologically, the hippocampal recall process in primates (Rolls and Xiang, 2006). We used a visual object-place memory task because this is prototypical of episodic memory. It has been shown that a one-trial odor-place recall memory task is hippocampal-dependent in rodents (Day et al., 2003). We designed a one-trial object-place recall task, in which the whole memory was recalled from a part of it. The task is illustrated in Figure 5 and described in its legend. Images of new objects were used each day, and within a day the same objects were used, so that with non-trial unique objects within a day, the recall task is quite difficult.

Recordings were made from 347 neurons in the hippocampus of a macaque performing the object-place recall task. The following types of neurons were found in the task (Rolls and Xiang, 2006).

**Figure 6:**
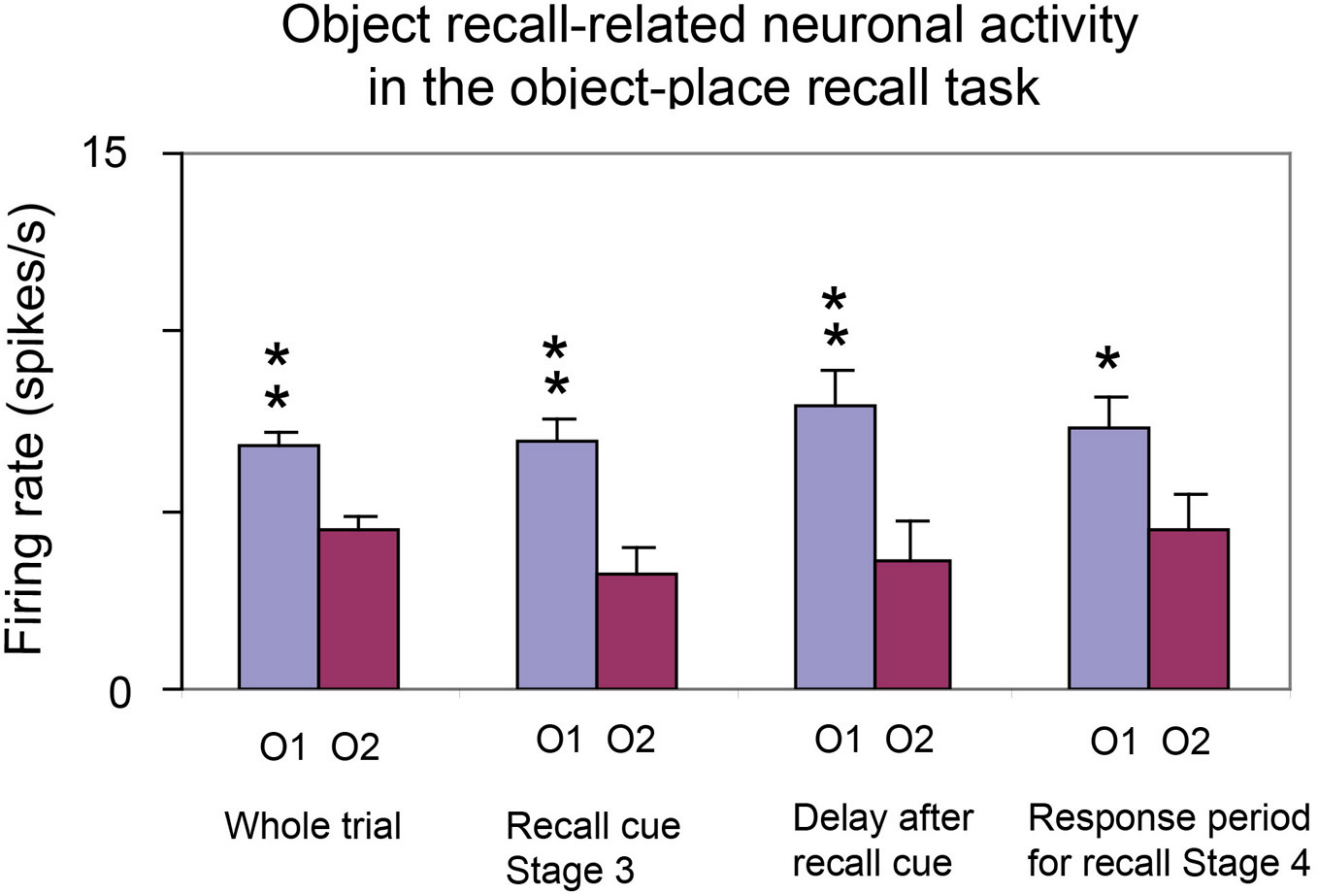
**Activity of a neuron with responses related to one of the objects used in the object-place recall task**. The firing rate to object 1 (O1) and object 2 (O2) are shown (mean firing rate in spikes/s across trials ± sem). The first histogram pair (on the left) shows the responses to the two objects measured throughout the trial whenever object 1 or object 2 was on the screen. The second histogram pair shows the neuronal responses when the objects were being shown in stage 3 as the recall cue. The third histogram pair shows the neuronal responses in the 0.5 s delay period after one of the objects had been shown in stage 3 as the recall cue. The neuron continued to respond more after object 1 than after object 2 had been seen, in this recall period in which the place was being recalled from the object. The fourth histogram pair shows the neuronal responses in stage 4 when the macaque was recalling and touching the place at which the cue recall object had been shown. The responses of the neuron were object-related even when the object was not being seen, but was being used as a recall cue, in the delay after stage 3 of the task, and in stage 4. ^**^p < 0.01; ^*^p < 0.05 [After Rolls and Xiang (2006)].

One type of neuron had responses that occurred to one of the objects used in the task. A number of these neurons had activity that was related to the recall process. An example of one of these neurons is shown in Figure 6. The neuron had activity that was greater to object one not only when it was shown in stages 1, 2, and 3 of the task, but also in the delay period following stage 3 when the object was no longer visible, and in stage 4, when also the object was no longer visible and the macaque was touching the remembered location of that object. Thus, while the location was being recalled from the object, this type of neuron continued to respond as if the object was present, that is it kept the representation of the object active after the object was no longer visible, and the place to touch was being recalled. Sixteen of the neurons responded in this way, and an additional 6 had object-related firing that did not continue following stage 3 of the task in the recall period. The difference of the firing rates of these 22 neurons to the different objects was in many cases highly statistically significant (e.g., *p* < 10^−6^; Rolls and Xiang, 2006). None of these neurons had differential responses for the different places used in the object-place recall task.

**Figure 7:**
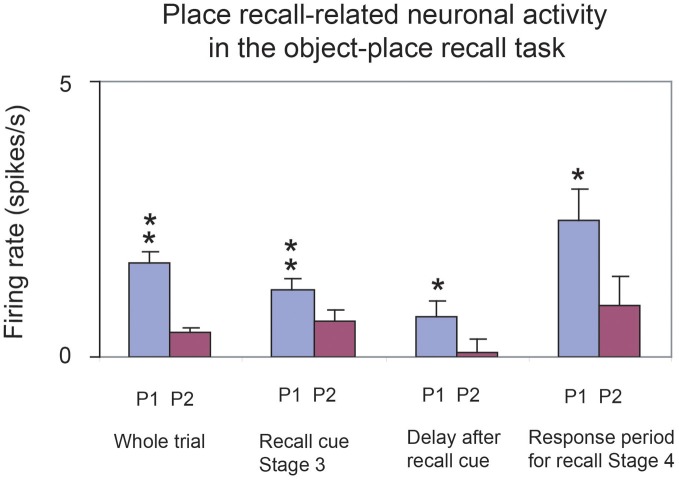
**Activity of a neuron with responses related to the left place (P1) in the object-place recall task**. The firing rate to place 1 (P1) and place 2 (P2) are shown (mean firing rate in spikes/s across trials ± sem). The first histogram pair (on the left) shows the responses to the two places measured when a stimulus was on the screen in stage 1 or stage 2. The second histogram pair shows the neuronal responses when the objects were being shown in stage 3 as the recall cue, and depending on whether the place to be recalled was place 1 or place 2. The third histogram pair shows the neuronal responses in the 0.5 s delay period after one of the objects had been shown in stage 3 as the recall cue. The neuron responded more when place 1 was the correct place to be recalled on a trial. The fourth histogram pair shows the neuronal responses in stage 4 when the macaque was recalling and touching the place at which the cue recall object had been shown. The responses of the neuron were place-related even in stage 3 when the object being shown as a place recall cue was at the top of the screen, in the delay after stage 3 of the task, and in stage 4. ^**^p < 0.01; ^*^p < 0.05 [After Rolls and Xiang (2006)]. (recallplace2.eps).

A second type of neuron had responses related to the place (left or right) in which an object was shown in stages 1 or 2 of each trial. An example of one of these neurons is shown in Figure 7. The neuron responded more when an object was shown in the left position (P1) than in the right position (P2) on the screen. Interestingly, when the recall object was shown in stage 3 of the trial in the top center of the screen, the neuron also responded as if the left position (P1) was being processed on trials on which the left position had to be recalled. This firing continued in the delay period after the recall cue had been removed at the end of stage 3, and into stage 4. Thus, this type of neuron appeared to reflect the recall of the position on the screen at which the object had been represented. Analysis of trials on which errors were made indicated that the responses were not just motor response related, for if due to some response bias the monkey touched the incorrect side, the neuron could still respond according to the correct recalled location. [On these error trials, therefore, the neuronal responses reflected the correct memory, and not the incorrect motor response—see further Rolls and Xiang (2006)]. Thirteen neurons had differential responses to the different places P1 and P2, and continued to show place-related activity in the recall part of the task, stage 3. Five other neurons had left-right place-related responses without a memory recall component, in that they did not respond in stage 3 of the task, when a non-spatial recall stimulus was being shown, and a place should be being recalled (see Table 1). The population of 18 neurons as a population had statistically significant place-related responses (Rolls and Xiang, 2006). The new finding is that 13 of the neurons had place-related responses when a place was being recalled by an object cue.

**Table 1:** **Numbers of neurons in the hippocampus with different types of response during the object-place recall task**.

Object with activity continuing after the recall cue	16
Object with activity not continuing after the recall cue	6
Place with activity during and after the recall cue	13
Place with activity during the recall cue	5
Object × Place	3
Total	347

The responses of the population of neurons recorded in one macaque are shown in Table 1. In addition to the neurons described above, 3 further neurons responded to particular combinations of objects and places, e.g., to object 1 when it was shown in place 1, but not to other combinations.

The recording sites of the object and of the place neurons were within the hippocampus proper (Rolls and Xiang, 2006). The mean firing rate of the population of responsive neurons (see Table 1) to the most effective object or place was 7.2 ± 0.6 spikes/s (±sem), and their mean spontaneous rate was 3.2 ± 0.6 spikes/s.

These findings (Rolls and Xiang, 2006) are the first we know in the primate hippocampus of neuronal activity that is related to recall. It is particularly interesting that the neurons with continuing activity to the object after it had disappeared in the recall phase of the task could reflect the operation of the object-place recall process that is hypothesized to take place in the CA3 cells. By continuing to respond to the object while the place is being recalled in the task, the object-related neurons could be part of the completion of the whole object-place combination memory from an autoassociation or attractor process in CA3 (Rolls and Kesner, 2006). Consistent with these findings, and with the computational theory, it has now been reported that human hippocampal neurons are activated during recall (Gelbard-Sagiv et al., 2008).

The neurons with recall-related activity in the object-place recall task also provide neurophysiological evidence on the speed of association learning in the hippocampal formation. Given that this is a one-trial object-place recall task, with the association between the object and its place being made in stages 1 and 2 of each trial (see Figure 5), it is clear that it takes just one-trial for the object-place associations to be formed that are relevant to the later recall on that trial. This is the speed of learning that is required for episodic memory, and this neurophysiological evidence shows that this type of rapid, one-trial, object-place learning is represented in the primate hippocampus.

### Reward-place neurons in the primate hippocampus

The primate anterior hippocampus (which corresponds to the rodent ventral hippocampus) receives inputs from brain regions involved in reward processing such as the amygdala and orbitofrontal cortex (Pitkanen et al., 2002). To investigate how this affective input may be incorporated into primate hippocampal function, Rolls and Xiang (2005) recorded neuronal activity while macaques performed a reward-place association task in which each spatial scene shown on a video monitor had one location which if touched yielded a preferred fruit juice reward, and a second location which yielded a less preferred juice reward. Each scene had different locations for the different rewards. Of 312 hippocampal neurons analyzed, 18% responded more to the location of the preferred reward in different scenes, and 5% to the location of the less preferred reward (Rolls and Xiang, 2005). When the locations of the preferred rewards in the scenes were reversed, 60% of 44 neurons tested reversed the location to which they responded, showing that the reward-place associations could be altered by new learning in a few trials. The majority (82%) of these 44 hippocampal reward-place neurons tested did not respond to object-reward associations in a visual discrimination object-reward association task. Thus, the primate hippocampus contains a representation of the reward associations of places “out there” being viewed, and this is a way in which affective information can be stored as part of an episodic memory, and how the current mood state may influence the retrieval of episodic memories. There is consistent evidence that rewards available in a spatial environment can influence the responsiveness of rodent place neurons (Hölscher et al., 2003; Tabuchi et al., 2003).

## Tests of the theory

### Dentate granule cells

The theory predicts that the dentate granule cell MF system of inputs to the CA3 neurons is necessary to store spatial memories, but not to recall them. Lassalle et al. (2000) have obtained evidence consistent with this in rats with damage to the MF system (Lassalle et al., 2000), and there is further evidence consistent with this (Lee and Kesner, 2004; Rolls and Kesner, 2006; Daumas et al., 2009).

The theory predicts that pattern separation is performed by the dentate granule cells. Evidence consistent with this has been found neurophysiologically in the small sparsely encoded place fields of dentate neurons (Jung and McNaughton, 1993; Leutgeb and Leutgeb, 2007) and their reflection in CA3 neurons (Leutgeb and Leutgeb, 2007). It has been shown that selective dentate lesions in rats (Gilbert et al., 2001; Gilbert and Kesner, 2003; Rolls and Kesner, 2006; Goodrich-Hunsaker et al., 2008; Rolls, 2008; Hunsaker and Kesner, 2013; Kesner, 2013) or dentate NMDA receptor knockouts in mice (McHugh et al., 2007) impair spatial, object-place (or reward-place: remembering where to find a reward) association tasks especially when the places are close together and require pattern separation before storage in CA3.

If adult neurogenesis in the DG does prove to be functionally relevant, its computational role could be to facilitate pattern separation for new patterns, by providing new dentate granule cells with new sets of random connections to CA3 neurons. Consistent with the dentate spatial pattern separation hypothesis (Rolls, 1989b,c; Treves and Rolls, 1992, 1994; Rolls, 1996b), in mice with impaired dentate neurogenesis, spatial learning in a delayed non-matching-to-place task in the radial arm maze was impaired for arms that were presented with little separation, but no deficit was observed when the arms were presented farther apart (Clelland et al., 2009). Consistently, impaired neurogenesis in the dentate also produced a deficit for small spatial separations in an associative object-in-place task (Clelland et al., 2009).

The theory predicts that the direct pp input from the entorhinal cortex to the CA3 cells (which bypasses the dentate granule cells) is involved in the recall of memory from the CA3 system, and Lee and Kesner (2004) have obtained evidence consistent with this in a Hebb–Williams maze recall task (Lee and Kesner, 2004).

### CA3

The theory predicts that the CA3 is especially important in object-place or reward-place tasks in which associations must be formed between any spatial location and any object (referred to as **arbitrary associations**). There is much evidence from subregion analyses involving disruption of CA3 that CA3 is necessary for arbitrary associations between places and objects or rewards (Gilbert and Kesner, 2003; Rolls and Kesner, 2006). Similar impairments were obtained following deletion of CA3 NMDA receptors in mice in the acquisition of an odor-context paired associate learning task (Rajji et al., 2006). If place or time is not a component, associative tasks such as odor-object association are not impaired (Rolls and Kesner, 2006), underlining the fact that the hippocampus is especially involved in episodic types of associative memory which typically involve place and/or time.

The theory predicts that the CA3 is especially important in object-place or reward-place **completion** tasks, in which associations must be completed from a part of the whole. It has been shown that if completion from an incomplete cue is needed, then CA3 NMDA receptors are necessary (presumably to ensure satisfactory CA3-CA3 learning) even in a reference memory task (Nakazawa et al., 2002; Gold and Kesner, 2005).

The theory predicts that the CA3 system is especially needed in **rapid, one-trial object-place, learning, and recall**. It has been shown that hippocampal NMDA receptors (necessary for Long-Term Potentiation to occur) are needed for one-trial flavor-place association learning, and that hippocampal AMPA/kainate receptors are sufficient for the recall, though the hippocampal subregion involved was not tested (Day et al., 2003). In subregion studies, Kesner and colleagues have shown that CA3 lesions produce chance performance on a one-trial object-place recall task (Kesner et al., 2008) and other object-spatial tasks (Kesner and Rolls, 2001; Rolls and Kesner, 2006). For example, CA3 lesions produced chance performance on both a one-trial object-place recall and place-object recall task (Kesner et al., 2008). This is evidence that CA3 supports arbitrary associations as well as episodic memory based on 1-trial learning. A control fixed visual conditional to place task with the same delay was not impaired, showing that it is recall after one-trial (or rapid, episodic) learning that is impaired (Kesner et al., 2008). CA3 NMDA receptors are as predicted by the theory necessary for rapid/one-trial spatial learning, as shown by a mouse knockout study by Nakazawa and colleagues (Nakazawa et al., 2003, 2004; Tonegawa et al., 2003). As described in section Systems-Level Neurophysiology of the Primate Hippocampus, we have shown that hippocampal CA3 neurons reflect the computational processes necessary for one-trial object-place event memory, used as a model for episodic memory (Rolls and Xiang, 2006).

Another type of test of the autoassociation (or attractor) hypothesis for CA3 has been to train rats in different environments, e.g., a square and a circular environment, and then test the prediction of the hypothesis that when presented with an environment ambiguous between these, that hippocampoal neurons will fall in an attractor state that represents one of the two previously learned environments, but not a mixture of the two environments. Evidence consistent with the hypothesis has been found (Wills et al., 2005). In a particularly dramatic example, it has been found that within each theta cycle, hippocampal pyramidal neurons may represent one or other of the learned environments (Jezek et al., 2011). This is an indication, predicted by Rolls and Treves (1998), that autoassociative memory recall can take place sufficiently rapidly to be complete within one theta cycle (120 ms), and that theta cycles could provide a mechanism for a fresh retrieval process to occur after a reset caused by the inhibitory part of each theta cycle, so that the memory can be updated rapidly to reflect a continuously changing environment, and not remain too long in an attractor state.

The theory predicts that if primates including humans can form an episodic memory in which objects or people are seen at particular locations even though the observer viewing the space has never been to those locations “out there” in space, there should be a neural system in CA3 that can support such associations between places “out there” in a scene and objects. Exactly this is provided by the spatial view neurons Rolls and colleagues have discovered that are present in CA3 (Rolls et al., 1997a, 1998, 2005; Robertson et al., 1998; Georges-François et al., 1999; Rolls and Xiang, 2005, 2006). Place cells will not do for this type of episodic memory.

Many further tests of the theory are described elsewhere (Rolls and Kesner, 2006; Rolls, 2008; Kesner et al., 2012).

### Recall via CA1 to neocortex

The theory shows quantitatively, analytically, how memories could be retrieved from the hippocampus to the neocortex (Treves and Rolls, 1994), and this has been shown by simulation of the multistage hippocampal system including the entorhinal cortex, dentate, CA3, CA1, and return to the entorhinal cortex to recall the memory to be quantitatively realistic (Rolls, 1995).

Many further tests of the theory are described elsewhere (Rolls and Kesner, 2006; Rolls, 2008, 2010b; Kesner et al., 2012).

## Discussion

The present theory holds that the hippocampus is used for the formation of episodic memories using autoassociation. This function is often necessary for successful spatial computation, but is not itself spatial computation. Instead, I believe that spatial computation is more likely to be performed in the neocortex (utilizing information if necessary for the particular task recalled from the hippocampus). Consistent with this view, hippocampal damage impairs the ability to learn new environments but not to perform spatial computations such as finding one's way to a place in a familiar environment, whereas damage to the parietal cortex and parahippocampal cortex can lead to problems such as topographical and other spatial agnosias, in humans (see Kolb and Whishaw, 2003). This is consistent with spatial computations normally being performed in the neocortex. [In monkeys, there is evidence for a role of the parietal cortex in allocentric spatial computation. For example, monkeys with parietal cortex lesions are impaired at performing a landmark task, in which the object to be chosen is signified by the proximity to it of a “landmark” (another object; Ungerleider and Mishkin, 1982)].

A key aspect of the hippocampal theory, which relates it very closely to episodic memory, is that the hippocampal system has the dentate granule to MF to CA3 cell system, which with its sparse connectivity (46 synapses onto each CA3 neuron) has a randomizing effect on the representations set up in CA3, so that they are as different as possible from each other; and that this then enables each associative memory formed in the CA3 recurrent collaterals to be as different as possible from all the other event memories (see section Dentate Granule Cells). This is then the optimal situation for the CA3 recurrent collateral effect to operate, for it can then associate together the random set of neurons that are active for a particular event (for example an object in a particular place), and later recall the whole set from any part. It is because the representations in CA3 are unstructured, or random, in this way that large numbers of memories can be stored in the CA3 autoassociation system, and that interference between the different memories is kept as low as possible, in that they are maximally different from each other. This is the essence of the hippocampus as a memory system suitable for unstructured memories such as event or episodic memories, each of which should be kept as distinct from the others as possible.

In this theory, the entorhinal cortex grid cell to dentate/CA3 place cell transformation that can be achieved by competitive learning (Rolls et al., 2006) is necessary so that a sparse representation of a place with different representations for different places suitable for association with an object or reward can be made available for CA3. The grid cell representation does not have this property, in that the representations of different places by grid cells are too highly correlated to be useful in an associative memory (Rolls, 2008). The utility of the entorhinal grid cell system is likely to be its role in path integration (McNaughton et al., 2006): in the idiothetic (self-motion) update of location, where the location is represented as a series of ring attractors of different spatial sizes, with no beginning and no end to each ring continuous attractor. Because a ring is endless, and the packet of activity just keeps going round the ring as locomotion continues, the system can perform path integration beyond any known environment, and in the dark, where the spatial environmental cues are not known or cannot be seen, so allowing for example return to base.

Once place cell representations have been set up in the hippocampus, they form a continuous spatial attractor network because of associative synaptic modification during locomotion producing synapses that are stronger the nearer together two places are, and thus the more likely coactivity between CA3 pyramidal cells is (Stringer et al., 2002a, 2004, 2005; Stringer and Rolls, 2006; Rolls, 2008). Although this is a continuous representation, as it has to be because space is continuous, it is suitable for association with the representations of objects, which are discrete, to form an episodic memory (Rolls et al., 2002). An important property of this spatial system is that there can be many separate maps, or charts, each of a different environment, stored in the hippocampus (Battaglia and Treves, 1998), as each chart has low correlations with any other chart, due in part to the randomizing effect of the MFs. The essential property of the hippocampal CA3 system then may be that it is not specialized to perform the computations necessary for spatial navigation (McNaughton et al., 1991; O'Keefe, 1991; Burgess and O'Keefe, 1996; McNaughton et al., 1996), but instead is a memory system that inevitably contains some continuous representations of space because places, landmarks, etc., are being encoded as part of an episodic memory system, and space is inherently continuous. Having said this, the hippocampus according to the present (Rolls') theory does form memories of where objects, people, rewards, etc., have been found in space, and so may be useful when one is looking for a particular object or reward, for the place can be recalled from the hippocampal representation. Once recalled to the neocortex, the place may then be used in a neocortically organized system to enable actions to be performed to reach a place, which could involve spatial navigation, using landmark or body centered algorithms, but also actions such as getting on a boat or bus, taking a plane, etc.

The hippocampus is the only part of the brain I know with the type of sparse connectivity implemented in the MF to CA3 synaptic connections to set up randomly different representations. It sets up a fascinating comparison with the neocortex. In the neocortex, the connectivity is set up to emphasize feedforward connectivity from one area to the next that has large numbers of connections onto each neuron, in the order of several thousand. This enables the neocortex to form representations by competitive learning that represent combinations of the “features” present at an earlier stage, and which are separated apart in the space. Such a process operating over several stages of a hierarchy enables representations of different objects to be formed. Here the representations are not arbitrarily and randomly different, as in CA3, but instead represent converge of information over large information spaces (using many synapses onto each neuron) that together enables an object to be “diagnosed,” that is, represented, as in Rolls' theory of invariant visual object recognition in the ventral cortical visual stream (Rolls, 1992, 2008, 2009, 2012c; Wallis and Rolls, 1997; Rolls and Stringer, 2006). In the neocortical system the local recurrent collateral connections between nearby cortical pyramidal cells play a largely different role to that in the hippocampal theory. In the neocortex they implement short-term memory which provides the basis for planning and attention; and decision-making including categorization (Rolls, 2008; Rolls and Deco, 2010).

A theory closely related to the present theory of how the hippocampus operates has been developed by McClelland et al. (1995). It is very similar to the theory we have developed (Rolls, 1987, 1989a,b,c, 2008, 2010b; Treves and Rolls, 1992, 1994) at the systems level, except that it takes a stronger position on the gradient of retrograde amnesia, emphasizes that recall from the hippocampus of episodic information is used to help build semantic representations in the neocortex, and holds that the last set of synapses that are modified rapidly during the learning of each episode are those between the CA3 and the CA1 pyramidal cells, as described above (see Figure 1). It also emphasizes the important point that the hippocampal and neocortical memory systems may be quite different, with the hippocampus specialized for the rapid learning of single events or episodes, and the neocortex for the slower learning of semantic representations which may necessarily benefit from the many exemplars needed to shape the semantic representation.

We have developed an approach to the mechanisms of schizophrenia based on the stability of neuronal systems with stochastic dynamics (Loh et al., 2007; Rolls et al., 2008a; Rolls and Deco, 2011; Rolls, 2012b). The cognitive symptoms such as a failure to maintain attention are related to a decrease in the stability of prefrontal cortical networks related to lower firing rates produced by NMDA receptor down-regulation. The negative symptoms such as reduced emotion, motivation, and hedonia are related to a decrease in the firing rates of orbitofrontal cortex and related networks produced by NMDA receptor down-regulation. The positive symptoms of schizophrenia include bizarre trains of thoughts, hallucinations, and delusions (Liddle, 1987; Mueser and McGurk, 2004). Rolls et al. propose that owing to reduced currents through NMDAR-activated channels, the basins of attraction of the high-firing-rate attractor states are shallow (Rolls, 2005; Durstewitz, 2007; Loh et al., 2007) in the temporal lobe, which includes the semantic memory networks and the auditory association cortex. Because of the resulting statistical fluctuations in the states of the attractor networks, internal representations of thoughts and perceptions move too freely around in the energy landscape, from thought to weakly associated thought, leading to bizarre thoughts and associations, and to hallucinations. Such thoughts might eventually be associated together in semantic memory, leading to false beliefs and delusions (Rolls, 2005, 2008, 2012b).

In addition, Loh et al. (2007) propose that a reduction in GABA interneuron efficacy in schizophrenic patients may also contribute to the generation of positive symptoms: lower GABA-interneuron efficacy reduces the depth of the basin of attraction of the spontaneous state, making it more likely that a high firing-rate attractor state will emerge out of the spontaneous firing of the neurons. This type of instability in which a network jumps because of noise into a high firing rate state that is not triggered by an external input to the network contributes it is suggested to the positive symptoms of schizophrenia, including for example hallucinations, delusions, and feelings of lack of control or being controlled by others (Loh et al., 2007; Rolls et al., 2008a; Rolls, 2012b). Empirical evidence supports this computational proposal: markers indicating decreased inhibition by the GABA system are found in neocortical areas (Lewis et al., 2005) and in parts of the hippocampus (Benes, 2010).

In this paper we have thus seen that there is neurophysiological evidence that different representations of the type important in episodic memory, including object and place, and reward and place, are brought together in the primate hippocampus, and even that the whole representation can be completed from a partial retrieval cue in a one-trial object-place memory task. It appears to be a property of the hippocampus that it is involved in associations when one of the associates is place or time (Rolls and Kesner, 2006; Rolls, 2008; Rolls and Deco, 2010). We have moreover seen that the representation of space in the primate hippocampus provided by spatial view cells is appropriate for episodic memory in primates including humans, for it is a representation of space “out there,” which is prototypical of the spatial representation that is involved in episodic memory in primates including humans. We have also described and updated a theory of how the hippocampal system could implement episodic memory (Rolls, 1987, 1989a,b,c, 1990a,b, 1991, 1995, 1996b, 2008, 2010b; Treves and Rolls, 1991, 1992, 1994; Rolls and Treves, 1998; Rolls and Kesner, 2006; Rolls and Deco, 2010), and shown how there is now considerable empirical support for the theory (Rolls and Kesner, 2006; Rolls, 2008; Kesner et al., 2012; Rolls, 2012b).

### Conflict of interest statement

The author declares that the research was conducted in the absence of any commercial or financial relationships that could be construed as a potential conflict of interest.
